# Impact and cost-effectiveness of different vaccination strategies to reduce the burden of pneumococcal disease among elderly in the Netherlands

**DOI:** 10.1371/journal.pone.0192640

**Published:** 2018-02-09

**Authors:** Dominic Thorrington, Leo van Rossum, Mirjam Knol, Hester de Melker, Hans Rümke, Eelko Hak, Albert Jan van Hoek

**Affiliations:** 1 Respiratory Diseases Department, Public Health England, London, United Kingdom; 2 Vaccination Committee, Health Council of The Netherlands, The Hague, The Netherlands; 3 Centre for Infectious Disease Control, National Institute for Public Health and the Environment, Bilthoven, The Netherlands; 4 Consultant in Vaccinology, Bilthoven, The Netherlands; 5 Groningen Research Institute of Pharmacy, University of Groningen, Groningen, The Netherlands; 6 Department of Infectious Disease Epidemiology, London School of Hygiene & Tropical Medicine, London, United Kingdom; Universidade Nova de Lisboa Instituto de Higiene e Medicina Tropical, PORTUGAL

## Abstract

**Background:**

*Streptococcus pneumoniae* causes morbidity and mortality among all ages in The Netherlands. To reduce this burden, infants in The Netherlands receive the 10-valent pneumococcal conjugated vaccine (PCV10), but older persons are not targeted. We assessed the impact and cost-effectiveness of vaccination with 23-valent pneumococcal polysaccharide vaccine (PPV23) or 13-valent PCV (PCV13) among all those aged 60, 65 or 70 and/or in combination with replacing PCV10 with PCV13 in the infant vaccination programme.

**Methods:**

A static cost-effectiveness model was parameterized including projected trends for invasive pneumococcal disease (IPD) and hospitalised community acquired pneumonia (CAP). The different strategies were evaluated using vaccine list prices and a 10-year time horizon. Incremental cost-effectiveness ratios (ICER) were calculated with the current strategy (infant vaccination program with PCV10) as reference.

**Results:**

Compared to the reference, the largest impact on pneumococcal disease burden was projected with a combined use of PCV13 among infants and PPV23 at 60, 65 and 70 years, preventing 1,635 cases of IPD and 914 cases of CAP. The most cost-effective strategy was vaccinating with PPV23 at 70 years only with similar low ICERs at age 60 and 65. The impact of the use of PCV13 among infants depends strongly on the projected herd-immunity effect on serotype 19A. Vaccinating elderly with either PCV13 or PPV23 was dominated by PPV23 in all investigated scenarios, mainly due to the lower price of PPV23.

**Conclusion:**

Under the current assumptions, the best value for money is the use of PPV23 for elderly, with a single dose or at five year increment between age 60 to age 70.

## Introduction

*Streptococcus pneumoniae* is a gram-positive pathogenic bacterium of which more than 90 serotypes are known. It causes high morbidity and mortality worldwide [1,2]. The pathogen causes diseases of varying degrees of severity from otitis media to community-acquired pneumonia (CAP), with or without hospital admission, through invasive pneumococcal disease (IPD) such as meningitis, bacteraemic pneumonia and sepsis. Some individuals are at increased risk of severe infections (i.e. infants, immunocompromised individuals and the elderly).

Currently, the 10-valent pneumococcal conjugate vaccine (PCV10) is included in the National Immunization Program (NIP) of the Netherlands in a 2+1 schedule (2, 4 and 11 months of age). The 23-valent polysaccharide vaccine (PPV23) is recommended to a limited number of high-risk groups (patients with asplenia and patients with CSF leakage) [3]. Before the introduction of PCV10, the 7-valent conjugate vaccine (PCV7) was used.

Introduction of PCV7 and PCV10 in the NIP substantially reduced IPD incidence caused by serotypes included in PCV7 and PCV10 in vaccinated as well as unvaccinated age groups [4–6]. However, the reduction of vaccine type IPD was partly offset by an increase of non-vaccine serotypes (NVT) and overall IPD decreased by about 20% in the elderly [6]. Therefore, there remains a burden of pneumococcal disease, especially among the elderly.

Recently, the results of a large phase 3 clinical trial in The Netherlands were presented [7]. In this trial the 13-valent pneumococcal conjugate vaccine (PCV13) was tested among persons 60 years and over. The results showed statistically significant protection of PCV13 against IPD and hospitalised vaccine type pneumococcal CAP [7].

The question under consideration in this analysis is whether to change the current strategy not to vaccinate the elderly population (60+ years) against pneumococcal infections. Possible new strategies include vaccination of elderly with PPV23 and/or PCV13 at age 60 years or at older age with or without revaccination. Furthermore, the infant immunisation schedule could be changed to administer PCV13 instead of PCV10.

We investigated the impact and cost-effectiveness of these different vaccination strategies. We projected future pneumococcal disease incidence specifically for vaccine serotypes and non-vaccine serotypes, including replacement of the non-vaccine serotypes. We estimated the potential impact of vaccinating different cohorts in terms of health-related quality of life and the economic burden caused by both IPD and hospitalised CAP from a health care provider’s perspective.

## Methods

A static cost-effectiveness model quantifying the impact of different vaccination strategies from a health care provider’s perspective was parameterized using surveillance data, literature values and expert opinion in case no consistent information was available.

### Invasive pneumococcal disease incidence

Estimates of the annual IPD incidence was obtained from the Dutch national surveillance covering approximately 25% of the Dutch population [4,5]. Data were available for the calendar years 2004 to 2015, split by age-group (60–64, 65–69, 70–74, 75–79, 80+ years), and split by the vaccine serotypes; these were the PCV7, PCV10 minus PCV7, PCV13 minus PCV10, PPV23 minus PCV13 and Non Vaccine Types (NVT). The incidence of disease after 2015 was projected using the following assumptions:

1. The IPD incidence caused by the PCV7-serotypes remains stable at its currently low incidence rate with continuing routine infant vaccination.
2. Given that PCV10 was introduced in 2011 we assume that the IPD incidence caused by the PCV10 minus PCV7 serotypes will decline further over a period of 5 years until the same relative indirect impact compared to PCV7 is reached; PCV7 dropped 90% from ~25 per 100.000 to ~2.5 per 100.000, thus PCV10 minus PCV7 serotypes are assumed to drop from 8.0 to 0.8 per 100.000.
3. The IPD incidence caused by the PCV13 minus PCV10 serotypes (3, 19A and 6A) remains stable when the administered vaccine within the infant programme remains PCV10, as observed in recent years in the Netherlands. However, when a PCV13 infant vaccination programme is introduced, the incidence (among those age 60+) will likely decline although this decline is less compared to what is assumed for PCV7 and PCV10 serotypes. Post-vaccination surveillance in countries using PCV13 show almost no indirect effect on serotype 3 as well as a ~40% impact on serotype 19A, and the incidence of IPD caused by serotype 6A was already reduced after the introduction of PCV7 [8]. Hence, no further decline in serotype 6A, no decline in serotype 3 and a 40% reduction of serotype 19A was applied. This eventually resulted in a drop of the incidence from 9.4 to 6.9 per 100.000 over a period of 7 years. A sensitivity analysis including a 90% drop of serotype 19A was included.
4. Due to serotype replacement the incidence of IPD caused by the PPV23 minus PCV13 serotypes will continue to increase with the same rate as they have been doing since the introduction of PCV7 (projected by a linear trend; see S1 Fig).
5. As the PPV23 minus PCV13 serotypes, the IPD incidence caused by non PPV23 serotypes continues to increase with the same rate as they have been doing since the introduction of PCV7 (projected by a linear trend; see S1 Fig).
6. The overall incidence of IPD will continue to increase, by a linear trend as per PPV23 and non PPV23 serotypes, until the overall incidence of IPD reaches 54 per 100,000 among those aged 60+, as this was roughly the pre-PCV-vaccination incidence rate in 2005–2006.

### All-cause community-acquired pneumonia

The majority of pneumonias in the hospital are recorded as “all-cause pneumonia without known aetiology” (ICD-10 code J18). Therefore, it is unknown what the contribution is of *Streptococcus pneumoniae*, or the pneumococcal serotypes that are included in the vaccine. To include the same disease dynamics as projected for IPD it was assumed that 30% of the pneumonia cases were caused by *Streptococcus pneumoniae* [9]. In case the overall incidence of IPD was projected to go up due to the ongoing serotype replacement, the etiological fraction of pneumonia caused by the pneumococcus increased as well given by an increasing percentage of all cause pneumonia caused by *Streptococcus pneumoniae*.

The pneumonia incidence (ICD-10 J18) at baseline was obtained from the Dutch hospital surveillance for the calendar year 2014.

For clarity, since the role of *Streptococcus pneumoniae* in pneumonia as diagnosed by the general practitioner (GP) is thought to be small and the potential effectiveness of the vaccines are unknown for primary-care treated pneumonia, we did not include this outcome in our projections.

### Vaccine effectiveness against IPD and pneumococcal pneumonia

**Fig 1:**
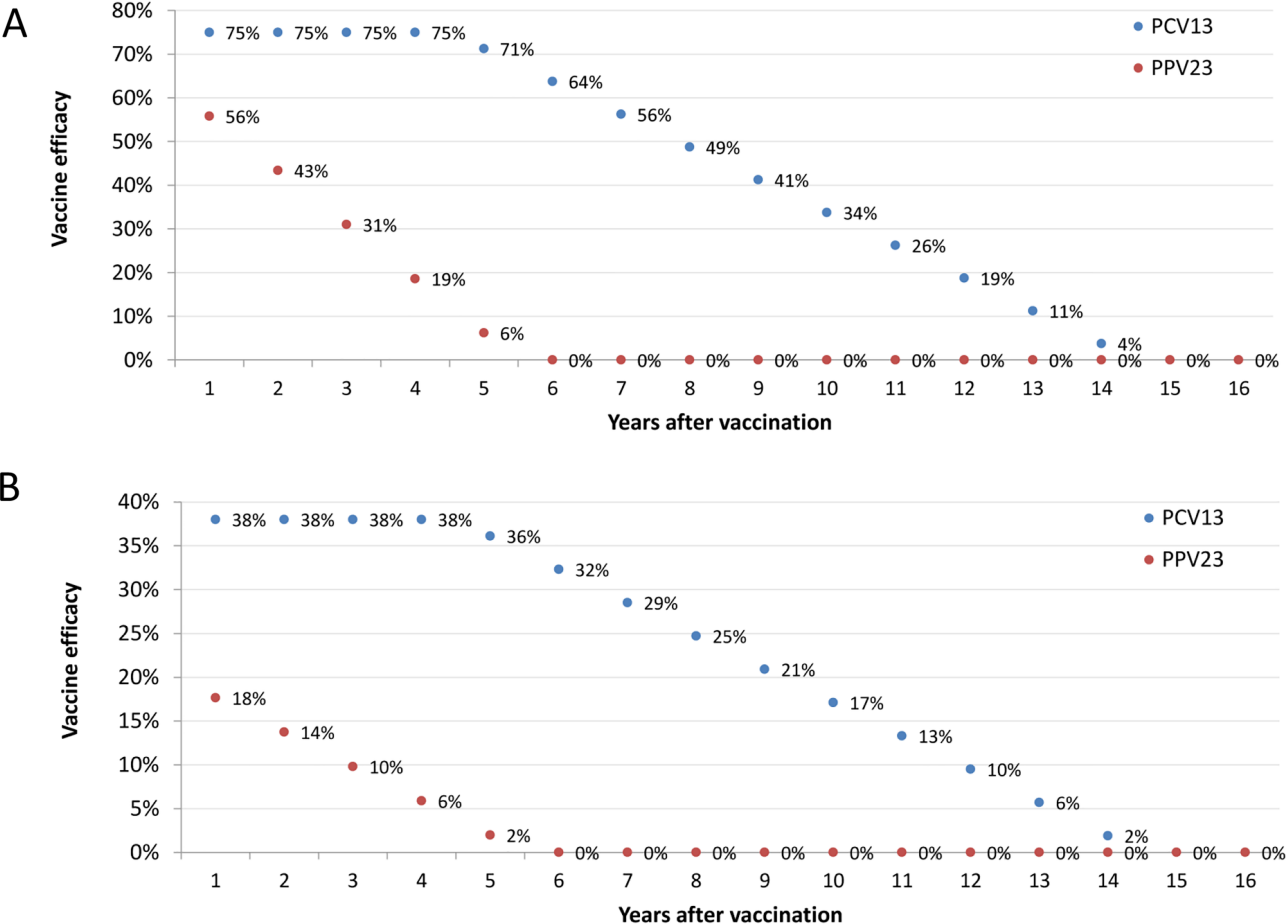
The assumed vaccine effectiveness against IPD (panel A; independent of age) and the vaccine effectiveness against vaccine type hospitalised CAP (panel B; independent of age).

For IPD the applied vaccine effectiveness against vaccine serotypes after vaccination was assumed to be 75% for PCV13 based on the phase 3 trial [7] and 62% for PPV23 based on a meta-analysis of several trials and cohort studies[10] at the start of first administration of the vaccine. We projected the respective efficacies to decline with different patterns over time. For PPV23 the effectiveness dropped linear, to 0% after 5 years, in line with the observed drop in vaccine effectiveness several years after vaccination [11]. For PCV13 a longer duration of protection was assumed as the conjugate in PCV13 induces T-cell immunity. The protection was kept stable for the first 4 years, as observed in the CAPITA trial, after which the effectiveness dropped linearly to 0% after 15 years of receiving the vaccine. See Fig 1 and Table 1.

**Table 1:** Main inputs for the cost-effectiveness analysis.

Parameter	Value	Reference
*QALY loss*		[13]
IPD	0.0709	
CAP	0.0709	
*Vaccine list price per dose*		[14]
PCV13	€ 72.67	
PPV23	€ 21.20	
PCV10	€ 60.56	
*Health care costs*		[13]
IPD	€ 14,584	
CAP	€ 7,872	
Mortality IPD	60–64;65–69;70–74;75–79;80+	RIVM surveillance data
PCV7 types	11%; 16%; 27%; 25%; 27%	
PCV10 minus PCV7 types	2%; 0%; 6%; 9%; 26%	
PCV13 minus PCV10 types	11%; 16%; 25%; 17%; 24%	
PPV23 minus PCV13 types	11%; 10%; 10%; 13%; 22%	
Non vaccine types	12%; 22%; 22%; 13%; 19%	
Mortality CAP	60–69 ; 70–79; 80–89; 90+	[12]
	9.6%; 13.9% ; 19.1% ; 25.5%	
*Discounting*		[15]
Costs	4%	
Health effects	1.5%	
*Vaccine* effectiveness		
PCV13 (elderly) against IPD	75% (41%-91%)	[7]
PCV13 (elderly) against VT pneumonia	38% (14%-55%)	[7]
PPV23 against IPD	64% (45%-75%)	[10]
PPV23 against VT pneumonia	19.6% (7.3%-28%)	See text
Coverage of vaccine ; elderly	50%	
Infants	100%	
Population size:		
1 year old	170,640	
60 years old	217,979	
65 years old	201,381	
70 years old	155,775	

For VT CAP the vaccine effectiveness for PCV13 was based on the modified intention to treat estimate in the CAPITA trial (38% [7]) with the same duration of protection as against IPD. There are no good estimates of the vaccine effectiveness of PPV23 against CAP. Therefore we used a similar overall effectiveness on all-cause CAP as PCV13 but with a shorter duration of protection. In the CAPITA trial all-cause pneumonia was reduced by 5% due to a 38% reduction of vaccine type CAP. Therefore, applying a back calculation, assuming that 30% of all cause CAP was caused by *Streptococcus pneumoniae* this reduction can only be observed when 44% of pneumococcal CAP was caused by PCV13 serotypes. This 44% share of PCV13 serotypes is similar to the 41% observed in the Dutch national surveillance in 2013, during the last year of the CAPITA trial [7]. Therefore, using the contribution of PPV23 in 2013 of 85% in Dutch surveillance data, the vaccine effectiveness against vaccine type CAP needs to be 19.6% to achieve a 5% reduction in overall all cause pneumonia. This 19.6% was used as the effectiveness of PPV23 at vaccination, but assumed to decline similarly as the effectiveness of PPV23 against IPD. The confidence interval around 19.6% was obtained by repeating the back calculation for both extremes of the confidence interval around 38%. Due to replacement it was assumed that replacing PCV10 with PCV13 among infants would not lead to an impact on overall hospitalised CAP among elderly.

### Mortality and life expectancy

Thirty-day mortality due to IPD was age-group and vaccine-type specific, using Dutch surveillance data from 2008–2012 [4]. Life-expectancy was calculated from the survival curve, and expected life years were corrected for background quality of life to obtain the quality of life adjusted life-expectancy. The age-specific mortality for CAP was obtained from a large database in Germany [12] as the age distribution was not available for the Netherlands.

### Costs and QALY loss

Based on a previous cost-effectiveness study in the Netherlands the costs included in this model are €14,584 for IPD and €7,872 for pneumonia [13]. The QALY loss per episode were estimated as 0.0709 for IPD and 0.0709 for CAP [13]. This value corresponds to a full reduction in quality of life for the duration of 26 days (365*0.0709) in a normally healthy person. The costs of the vaccines per dose were set at €60.56 for PCV10, €72.67 for PCV13 and €21.20 for PPV23 [14] based on the list price, excluding additional administration costs.

### Model

The impact of vaccination was quantified by comparing the difference in future disease incidence, and subsequent mortality, costs and loss of quality of life, by projecting the future with the new vaccination strategy under study and comparing to the reference current strategy.

Disease incidence by age was projected using incidence estimates for five different age groups (60–64, 65–69, 70–74, 75–79 and 80+) where people in the model aged by year and had a probability to acquire disease based on the incidence corresponding to their age-group. Vaccine effectiveness was applied such that the incidence was reduced corresponding to the vaccine effectiveness by year since vaccination (see the paragraph on vaccine effectiveness above and S1 File; incidence with vaccination = incidence without vaccination * (1-VE)). Strategies with doses at different ages were implemented such that each age group received the dose from day one of the programme.

Background mortality based on national statistics was applied to obtain realistic aging in the population, and to calculate the life years lost in case of death using the life-expectancy. In the model, based on the uptake of influenza vaccine, a conservative 50% uptake was applied to generate results for vaccination of elderly with PCV13 and PPV23. The coverage did not affect the cost-effectiveness results due to the linear link between impact and costs of the programme. It was assumed that there is a high vaccination coverage (100%)of the infant programme [6].

Vaccination strategies were modelled to be introduced in 2018 and the projected costs and QALYs were discounted to this year. As the study population was of or above retirement age and the cost-effectiveness analysis was performed from a healthcare provider perspective, no additional societal costs were included in our model on top of healthcare costs. The relation between the costs of the programme and the benefits were expressed in an incremental cost-effectiveness ratio (ICER), using the current programme (vaccinating infants with PCV10) as the reference.

### Discounting and time horizon

The future costs and QALYs were discounted using a discount rate of 4.0% for costs and 1.5% for health benefits according to national guidelines [15]. Given the highly dynamic nature of the pneumococcus as well as the continuous development of new generations of vaccines the time horizon was set at 10 years. A longer (15 & 50 years), and shorter (5 years) time horizon was included in the sensitivity analysis. In the current analysis, two different vaccination approaches are compared: a cohort approach where people are vaccinated at a certain age and only those people receive the health benefits over time, compared with an infant vaccination approach where infants are vaccinated and all members of the population receive the health benefits through indirect protection against pneumococcal disease. Therefore, the benefits of these two approaches appear at different times in the future; the infant approach generates an almost immediate impact for all ages, whereas the impact of the cohort approach appears over time.

Therefore, within the time constraint of a 10-year time horizon indirect protection by vaccinating infants is incomparable to direct protection of vaccinating the elderly: vaccinating infants for 10 years results in an immediate effect within the 10 year period, whereas vaccinating a cohort of, for example, 60 year olds can generate health benefits after the 10 year period. Therefore, we decided to additionally (on top of the 5, 15 and 50 years’ time horizon) report an unlimited or life-time time horizon to (virtually) minimize the differences in temporal effects between direct and indirect protection. As this scenario allows for the inclusion of benefits after the 10-year period this should aid comparison with other models using a life-time time horizon.

### Sensitivity analysis

The sensitivity analysis tested the sensitivity of the ICER to assumptions on vaccine effectiveness, age at vaccination, mortality assumptions, cost assumptions, adding administration costs, QALY assumptions, discounting, time horizon and the level of herd protection generated by the infant programme.

## Results

### IPD disease in the future

**Fig 2:**
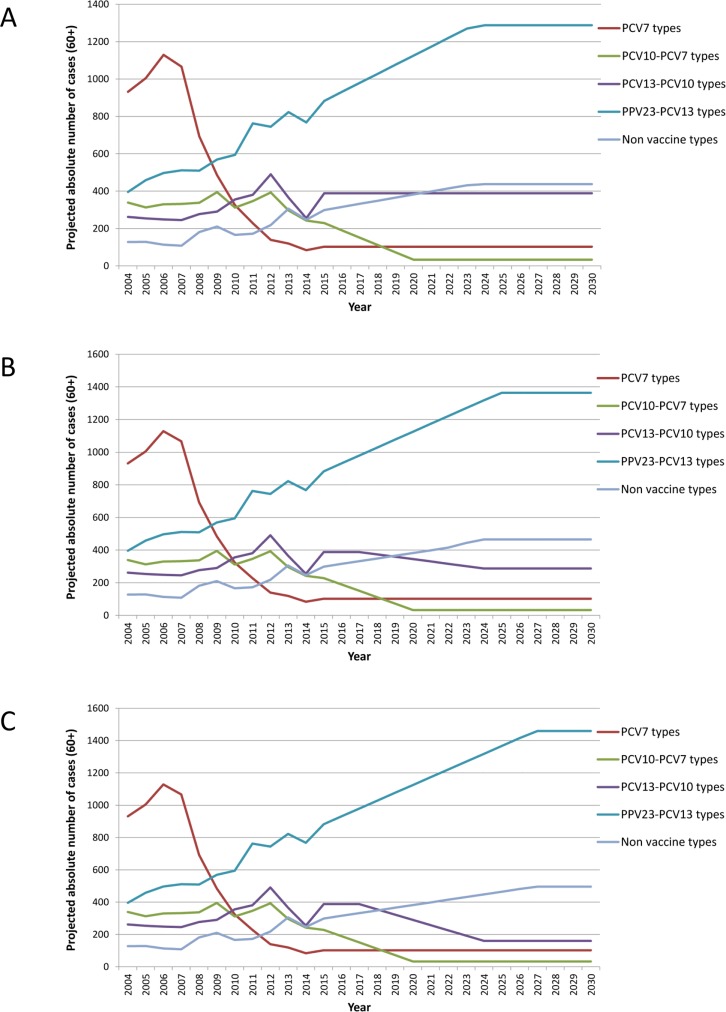
Projected cases of invasive pneumococcal disease under the different strategies. Panel A) current strategy of vaccinating infants with PCV10 including a herd effect for the PCV10 minus PCV7 serotypes and increase of the non-PCV13 serotypes. Panel B) with a strategy when PCV13 is introduced among infants, with a 40% herd protection effect against 19A, also including the indirect effects against PCV10 minus PCV7 serotypes and an increase in non-PCV13 serotypes. Panel C) for a strategy where PCV13 is introduced among infants with a 90% herd protection against 19A, also including the indirect effects against PCV10 minus PCV7 serotypes and an increase in non-PCV13 serotypes.

In Fig 2 the projected disease burden for the individuals aged 60 years and older is presented. There are two different scenarios resulting in 3 different projections: one with PCV10 among infants (including herd effects for the PCV10 minus PCV7 serotypes), one with PCV13 among infants including 40% herd protection against serotype 19A in those aged 60 years and above and one with PCV13 among infants including 90% herd protection against 19A in those aged 60 years and above.

In all scenarios, the steady state of IPD is 54 per 100,000 (the incidence pre-vaccination). The main impact of an infant vaccination strategy introducing PCV13 instead of PCV10 is a projected delay of approximately 2 years (40% reduction in 19A) or 4 years (90% reduction of 19A) in reaching the new steady state of 54 per 100,000.

### Additional budget impact of the different strategies

Of the three investigated strategies, vaccinating 50% of a cohort of 60 year olds (108,990 people) with PCV13 has the largest budget impact, with costs of € 7.92 million per year (assuming the list price of € 72.67). Vaccinating the same people with PPV23 will cost less i.e. € 2.31 million per year (assuming the list price of € 21.20); vaccinating a cohort of 1 year olds (170,640 infants) with 3 doses of PCV13 will cost € 6.2 million per year (assuming the extra costs of € 12.11 per dose of switching from PCV10 to PCV13).

### Prevented cases

Given an uptake of 50% and the relative short duration of (full) protection as well as the time window of 10 years the absolute impact of the different vaccination strategies exceeds the 1,000 prevented cases of IPD (Table 2) only in the strategies when there is a re-vaccination with PPV23 at five years interval, or under the assumptions that there is a large herd impact against 19A in case of an infant PCV13 programme, or when the infant PCV13 programme was combined with vaccinating with PPV23 at age 60, 65 and 70. The absolute impact of either the PCV13 or PPV23 strategies among elderly are similar preventing around the same number of deaths due to IPD (between 34 and 62 for PCV13 and 38 and 54 for PPV23 depending on age), and death due to hospitalised CAP (between 31 and 64 for PCV13 and 22 to 48 for PPV23 depending on age).

**Table 2:** The total number cases, deaths and QALYs for IPD and vaccine type CAP and the prevented cases, deaths, QALYS and costs for the different disease end points vaccination, among infants, at age 60 65 and 70, as well as the overall programme costs and the cost-effectiveness results over period of 10 years. Vaccination coverage 50% for both PCV13 and PPV23 and full coverage for PCV13 among children.

	PCV13 in infants (40% reduction 19A)	PCV13 in infants (90% reduction 19A)	PCV13 at 60	PCV13 at 65	PCV13 at 70	PPV23 at 60	PPV23 at 65	PPV23 at 70	PPV23 at 60+65+70	PCV13 in infants + PPV23 at 60+65+70
***All IPD***										
Cases ≥ 60	21,496	21,496	21,496	21,496	21,496	21,496	21,496	21,496	21,496	21,496
Cases Prevented	330	1,049	330	414	303	396	509	424	1,329	1,635
Deaths	3,193	3,193	3,193	3,193	3,193	3,193	3,193	3,193	3,193	3,193
Deaths Prevented	73	207	34	62	61	38	53	54	145	2,306
QALYs	26,193	26,193	26,193	26,193	26,193	26,193	26,193	26,193	26,193	26,193
QALY Gained (Q1)	584	1,679	474	719	558	579	672	545	1,796	2,306
Costs savings (C1)	€ 4,192,946	€ 12,867,806	€ 3,831,215	€ 4,860,402	€ 3,522,591	€ 4,752,538	€ 6,101,323	€ 5,089,019	€ 15,942,880	€ 19,848,727
***VT CAP admissions***										
Cases ≥ 60	16,404	16,404	16,404	16,404	16,404	52,977	52,977	52,977	52,977	52,977
Cases Prevented	0	0	348	449	507	246	318	376	940	914
Deaths	2,376	2,376	2,376	2,376	2,376	7,650	7,650	7,650	7,650	7,650
Deaths Prevented	0	0	31	44	64	22	28	48	98	96
QALYs	16,669	16,669	16,669	16,669	16,669	53,666	53,666	53,666	53,666	53,666
QALY Gained (Q2)	0	0	436	508	603	330	359	488	1,177	1,145
Savings (C2)	€ 0.00	€0.00	€ 2,186,616	€ 2,822,043	€ 3,192,853	€ 1,593,938	€ 2,059,962	€ 2,435,584	€ 6,089,484	€ 5,919,649
**Discounted costs and savings**										
Costs(C3)	€ 52,293,583	€ 52,293,583	€ 66,810,078	€ 61,722,828	€ 47,744,691	€ 19,490,487	€ 18,006,384	€ 13,928,546	€ 51,425,417	€ 103,719,000
Netto (= C3-C1-C2)	€ 48,100,637	€ 39,426,778	€ 60,792,248	€ 54,040,383	€ 41,029,247	€ 13,144,010	€ 9,845,099	€ 6,403,943	€ 29,393,053	€ 77,950,624
**QALYs gained (= Q1+Q2)**	584	1,679	910	1,227	1,161	909	1,031	1,033	2,973	3,451
***Costs per QALY***	€ 82,425	€ 23,485	€ 66,796	€ 44,028	€ 35,346	€ 14,452	€ 9,553	€ 6,201	€ 9,887	€ 22,588

### Cost-effectiveness of different pneumococcal vaccination strategies

To explore the cost-effectiveness of the proposed vaccination strategies we present several results in Table 2. Results are shown where a 10-year time horizon is applied. The most cost-effective strategy with a single vaccination is introducing PPV23 among those aged 70 (ICER € 6,201 per QALY). The least cost-effective scenario is vaccinating infants with PCV13 in case the herd immunity effects against 19A remains 40% as this scenario generates an ICER of € 82,425 per QALY. When the herd effects reach 90% against 19A the least cost-effective scenario is vaccinating the elderly with PCV13 at 60 years (ICER of € 66,796). Combining multiple vaccinations such as vaccinating at 60, 65 and 70 with PPV23 and vaccinating infants with PCV13 and elderly with PPV23 increases the health impact, but are not necessarily more cost-effective.

**Fig 3:**
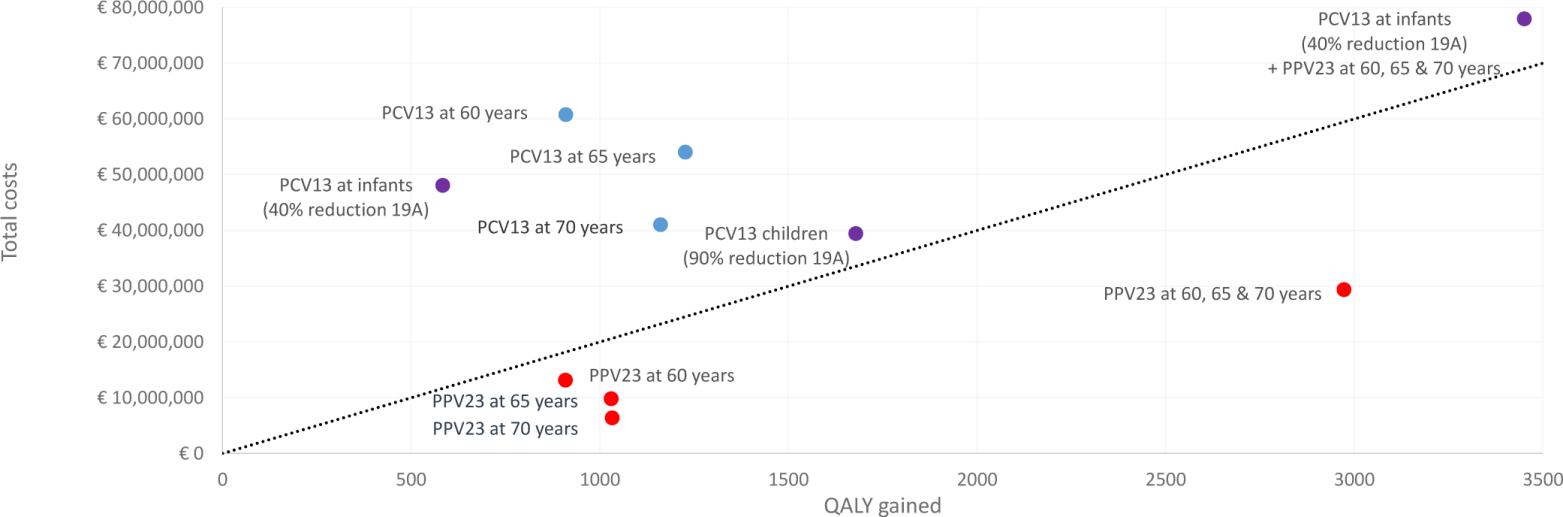
Cost-effectiveness plane for the presented scenarios (using a 10 year time horizon). The dotted line represents a cost-effectiveness threshold of €20,000 per QALY.

Fig 3 shows the cost-effectiveness plane (Fig 3). All PPV23 only strategies among older persons are cost-effective. Interestingly, vaccinating with one dose of PCV13 at age 65 and 70 could generate a larger health impact compared to vaccinating at the same age with PPV23, however vaccinating with PCV13 is more expensive, resulting in a higher ICER. The largest health benefit is estimated with the strategy where elderly are vaccinated once at age 60, 65 and 70 and at the same time the infants receive PCV13 instead of the current PCV10 (with the assumption of 40% herd protection against 19A).

As a 10-year time horizon was used instead of a life-time time horizon, some of the potential benefits were not included. Using an infinite time horizon while the programme last 10 years the ICER for PCV13 among those aged 60 drops from € 66,796 to € 33,419 per QALY, and the ICER for PPV23 from € 14,452 to € 12,191 per QALY (Table 3).

**Table 3:** ICER of the two cohort-approaches under 10-year implementation, using an infinite time horizon.

	ICER for PCV13	ICER for PPV23
At age 60	€ 33,419	€ 12,191
At age 65	€ 15,414	€ 3,195

### Sensitivity analysis

Table 4 describes the impact of changing the time-horizon. Expanding the time-horizon beyond 10 years increases the cost-effectiveness of the PCV13 strategy among elderly, as more positive effects of vaccination is included due to its relatively long duration of protection. Expanding the time horizon for PPV23 improves the cost-effectiveness as well. Due to replacement effects, the cost-effectiveness of vaccinating infants declines by expanding the time horizon, as after there is a full replacement there is no additional benefit of vaccinating the infants.

**Table 4:** Impact of time horizon on ICER in the scenario with replacement and vaccinating at 65 years of age.

Time horizon	5 years	10 years	15 years	50 years
***PCV13***				
At 65 years	€ 81,293	€ 44,028	€ 32,913	€ 18,281
***PPV23***				
At 65 years	€ 13,793	€ 9,553	€ 8,176	€ 5,258
**PCV13 among infants**				
	€ 79,744	€ 82,425	€ 95,770	€ 100,001

A head to head comparison (Table 5) of changing important assumptions between cohort vaccination with PCV13 and PPV23 reveals that compared to PCV13 PPV23 remains more cost-effective without an effect on CAP. Applying the lower and upper bound of the estimated vaccine effectiveness for PCV13 and PPV23 showed that the ICER for PPV23 is better in both scenarios compared to PCV13, and for PPV23 the ICER remains under €50,000 in all scenarios.

**Table 5:** Sensitivity analysis of important assumptions between PCV13 and PPV23 among elderly.

	PCV13 @ 60	PCV13 @ 65	PPV23 @ 60	PPV23 @ 65
Base case	€ 66,796	€ 44,028	€ 14,452	€ 9,553
Lower bound vaccine effectiveness for both IPD and CAP	€ 151,288	€ 98,552	€ 28,431	€ 20,618
Upper bound vaccine effectiveness for both IPD and CAP	€ 48,904	€ 32,221	€ 9,694	€ 5,734
No impact on pneumonia	€ 132,897	€ 79,033	€ 25,454	€ 17,714
Lower bound vaccine effectiveness for IPD + no impact pneumonia	€ 247,317	€ 148,658	€ 38,171	€ 27,836

The cost-effectiveness results are very sensitive to changes in the mortality rate and the total costs of implementing the programme (Table 6 and S2 Fig). The health care costs, discount rate for health effects, and the QALY weights for IPD and CAP did not have a large influence on the findings.

**Table 6:** Sensitivity analysis of the main input in the cost-effectiveness for the 3 strategies.

	PCV13 at 65	PPV23 at 65	PCV13 at infants (40% herd protection against 19A)
*Base case*	*€ 44*,*028*	*€ 9*,*553*	*€ 82*,*425*
No QALY loss IPD+CAP	€ 46,141	€ 10,085	€ 85,682
No mortality IPD	€ 101,020	€ 25,115	€ 2,168,086
No mortality CAP	€ 72,181	€ 14,207	No effect on CAP
No mortality at all	€ 961,290	€ 181,060	€ 2,168,086
Price per dose of other vaccine (€ 21,20 for PCV13/€ 72,67 for PPV23)	*€ 8*,*411*	€ 51,970	cost saving until €1.53 ; €4.2 extra for ICER €20,000
List price + €10 administration costs per dose	€ 50,948	€ 17,794	€ 156,421
Double costs IPD & CAP	€ 37,769	€ 1,634	€ 75,240
Half cost IPD & CAP	€ 47,157	€ 13,512	€ 86,017
Discounting both costs and health benefits with 4%	€ 61,369	€ 13,203	€ 107,435

## Discussion

This paper investigates three different vaccination strategies to prevent pneumococcal disease in elderly, focusing on a country with an existing infant vaccination programme using PCV10, but with no existing pneumococcal vaccination programme targeting the elderly. All three new vaccination strategies reduce the burden caused by the vaccine-serotypes among the elderly. However, our analysis showed that the most cost-effective strategy was vaccinating a cohort of elderly with PPV23, in a single dose or a multi-dose strategy, with comparable ICERs as for example HPV vaccination which was introduced in the NIP [16,17].

Each investigated strategy was at least in one of the (sensitivity) analyses cost–effective using a threshold of €20,000 per QALY (Tables 5 and 6). When the incidence of the PCV13 serotypes remains stable, as we projected, the use of PCV13 among elderly can be cost-effective especially in older age groups. However, as with the PPV23 strategy the impact on disease is limited to specific age groups. Vaccinating infants with PCV13 instead of PCV10 will affect all elderly individuals independent of age and is relatively cheap (even considering 3 doses among infants), as only the extra costs of PCV13 over PCV10 have to be budgeted. Depending on the time horizon and the herd immunity impact on 19A, vaccinating infants with PCV13 instead of PCV10 is a better option than vaccinating the elderly with PCV13. Importantly, vaccinating elderly with PPV23 was more beneficial in all investigated strategies. A PPV23 strategy is cheaper (1 dose for PPV23 versus 3 doses of PCV13 among infants) and generated more health benefit. Although our analysis was done in a country using PCV10 in childhood vaccination, the here described phenomenon in elderly can be translated to countries using PCV13 in their childhood programme.

Included replacement trends influenced the cost-effectiveness of all strategies to some extent, but especially the PCV13 strategy among infants and the PPV23 vaccination in elderly. The increase in non-PCV13 serotypes offsets the benefit of reducing the PCV13 serotypes by the infant vaccination strategy on the longer term, which makes vaccinating the infants less cost-effective, and the increase in the serotypes targeted by PPV23 makes vaccinating the elderly with PPV23 more cost-effective. This effect becomes more pronounced when longer time horizons were used.

Our results support a vaccination strategy including re-vaccination with PPV23. Revaccination with PPV23 is not without controversy, as receiving a second dose of PPV23 could actually give a lower immune response than after the first dose, so called hypo responsiveness. Recently, Remschmidt et al. [18] concluded, based on a review of the available studies, that although such effect is present in immunogenicity data in situations where there is a short interval (<1 year) between subsequent doses, no such effect is present in case this period is longer. This suggests that with at least 5 years between doses, as in our tested strategy, hypo-responsiveness should not be a major concern. Unfortunately, no studies with clinical end points are available, which merits careful surveillance if such strategy is implemented.

There are some important considerations in the interpretation of our results. The projections used in this work are simple and illustrative. They are however not based on a dynamic transmission model to predict the contributions of the different vaccine-serotypes in the future. There are some limitations with regard to the validity of our estimated projections. Firstly, the decline of the PCV10 serotypes can halt, not reaching the incidence of 0.8 per 100,000 in those 60 years and over. This scenario will imply that the indirect effects of the infant programme, for the three extra serotypes included in PCV10 will be different compared to those serotypes included in PCV7. Should this scenario happen this will leave more disease burden available to be reduced by PCV13 (given to elderly or infants) and PPV23. Secondly, PCV13 can have a different indirect effect from what we modelled, including more herd protection against serotype 3 or 6A. Thirdly, there is an uncertainty around the absolute incidence of IPD caused by the different serotypes. For example, when the absolute incidence of the PCV13 serotypes increases, the cost-effectiveness of vaccinating the elderly with PCV13 will improve. Fourthly, in our scenario with replacement by non-PCV13 serotypes we can have underestimated the speed with which the replacement happens. When this replacement is more rapid, the extra benefits of vaccinating the infants with PCV13 will be off-set earlier by the additional increase in non-PCV13 serotypes, making this scenario less cost-effective. Furthermore, the future price within the national immunisation programme is negotiated with manufacturers and is not publicly available. Therefore, the used list price is not the price that would be paid for any of these vaccines when introduced at national level. Also the interpretation that switching from PCV10 to PCV13 in routine childhood vaccination is relatively cheap as stated earlier, can be affected by this tendering process i.e it is expected that the negotiated price rises if only one vaccine is included in the tendering process. The used list price does also not include programmatic costs as reimbursement of the general practitioner to provide the dose, the call and recall of those eligible, promotional campaigns and surveillance costs. In case €10 programmatic costs is added to the price per dose the ICER for PPV23 remained below €20,000 Furthermore, there is no official cost-effectiveness threshold per QALY in the Netherlands which defines a result being cost-effective. In our graph a threshold of €20,000/QALY was applied, which has been proposed for preventive measures and used before in The Netherlands [19]. In addition, as infants and children are the main driver of pneumococcal transmission, in assessing the cost-effectiveness of an infant strategy the impact on the disease burden in the non-targeted age groups below 60 years should be included as well; these were omitted from our analysis for a fairer comparison between strategies to prevent disease among elderly. In The Netherlands, it is common to use a societal perspective which should include the impact on work loss. This was omitted in this analysis but could positively affect the cost-effectiveness result for the working population, thus when vaccinating at age 60.

Incremental cost-effectiveness ratios and the verdict of being “cost-effective” are extremely hard to compare within the international literature due to the different assumptions on discounting, time horizon, threshold price, vaccine price, absolute incidence of vaccine serotype related disease and assumed vaccine effectiveness parameters. Nevertheless, we compared our assumptions and conclusion with other studies. Our conclusion that given the high list price of PCV13 and/or the indirect effects of an infant strategy, PCV13 is not the most cost-effective strategy to be used among a population wide cohort of elderly is in line with recent studies from Germany [20], Belgium [21], US [22], UK [23] and Australia [24]. In some studies the importance to differentiate between different levels of risk in the population was highlighted [13,22], e.g. to focus on mid- to high risk groups instead of vaccinating everybody. This was not evaluated in our study. Regarding the question, which option is better to use among elderly, PCV13 or PPV23, the literature is undecided, as there are no head-to-head trials for these two options, necessitating comparing outcomes between different types of studies, in different countries, years and patient-groups. Especially the level and duration of protection of PPV23 against IPD and CAP are still important unknowns [21,24]. In our analysis, PPV23 was more cost-effective than PCV13 even in a conservative scenario where we assumed no protection of PPV23 against CAP.

In this analysis, we focussed on the consequences/results from a cost-effectiveness perspective. However, in view of considering possible changes in vaccination strategies towards elderly other factors also play a role. This includes the relatively low vaccine effectiveness in elderly resulting in a limited overall impact on pneumococcal disease, and the perceived disease risk and the related willingness of elderly to vaccinate against pneumococcal disease. Actually, elderly consider pneumococcal disease the most severe and most relevant disease to vaccinate against [25]. A discrete choice method estimated that 76% among elderly would take such vaccine [25] suggesting that the 50% uptake assumed in our analysis is conservative. In contrast, in the last years the uptake for the influenza vaccine, targeted at the same age groups, has declined in The Netherlands [26]. Therefore, an introduction of a strategy targeting this age should be accompanied by an education and information campaign.

In conclusion, the dynamics of circulating strains of pneumococci, with an increase in the circulation of serotypes which are not included in PCV13, complicates the elucidation of the best strategy to reduce the disease burden among the elderly. Considering a time horizon of ten years and best estimates of vaccine effectiveness for PPV23 as well as the indirect effects of PCV13 among infants, focussing on strategies with PPV23 seems the best option; providing vaccination at an individual age or by providing several doses given at five-year increments between 60 and 70.

## Supporting information

S1 Fig The fitted linear trend for the PPV23 minus PCV13 serotypes and non-PPV23 serotypes using the incidence per 100,000 for those aged 60 and above. (PDF)

S2 Fig Linear relationship between vaccine price and the ICER. (PDF)

S1 File Main formula in the static model. (PDF)
