# Supplementary material for: Impact and cost-effectiveness of different vaccination strategies to reduce the burden of pneumococcal disease among elderly in the Netherlands
Source: PLoS One. 2018 Feb 9;13(2):e0192640. doi: 10.1371/journal.pone.0192640 (PMC5806887; doi:10.1371/journal.pone.0192640)
Supplement: S1 Fig — (PDF) [file pone.0192640.s001.pdf]

Figure S1

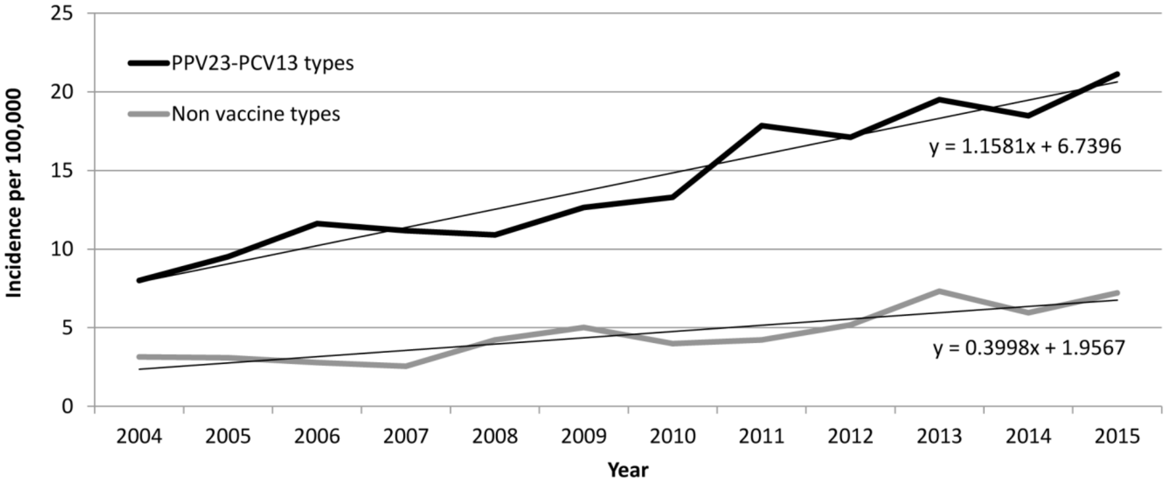

Figure S1 The fitted linear trend for the PPV23 minus PCV13 serotypes and non-PPV23 serotypes using the incidence per 100,000 for those aged 60 and above.
