# Supplementary material for: Impact and cost-effectiveness of different vaccination strategies to reduce the burden of pneumococcal disease among elderly in the Netherlands
Source: PLoS One. 2018 Feb 9;13(2):e0192640. doi: 10.1371/journal.pone.0192640 (PMC5806887; doi:10.1371/journal.pone.0192640)
Supplement: S2 Fig — (PDF) [file pone.0192640.s002.pdf]

Figure S2

The sensitivity of the ICER in respect to the price of the vaccine

To provide more insight on the impact of changing the price assumptions on the ICER we performed an additional analysis focusing on vaccinating those aged 65 with PPV23 or PCV13, or vaccination infants with PCV13 instead of PCV10. Fig S2 shows the linear relationship between vaccine price and the ICER. It can be seen that, to reduce the burden of disease among those 60 and over, vaccinating infants with PCV13 instead of PCV10 is only cost-effective when PCV13 has (almost) the same price as PCV10. When PCV13 is offered for the same price as PPV23, PCV13 is slightly the more cost-effective option as soon as the price per dose reaches €10 or above.

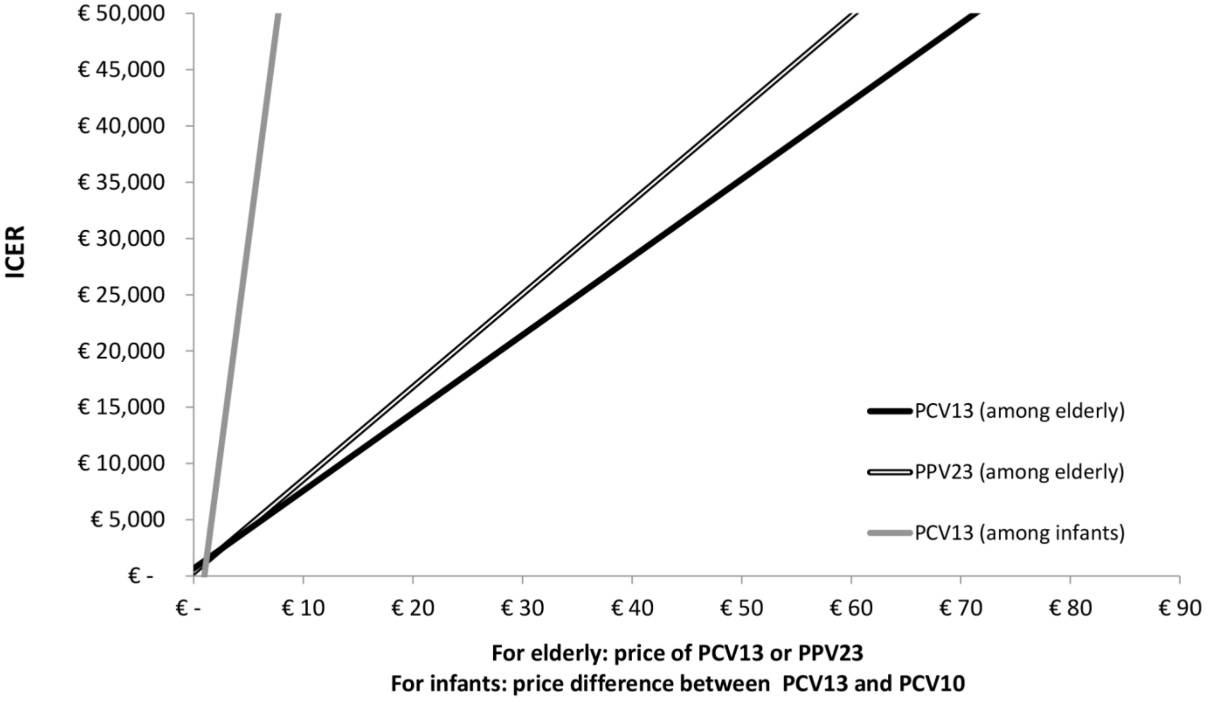

Fig S2. Linear relationship between vaccine price and the ICER. For vaccination of the elderly an additional €10 administration was assumed.
