# Supplementary material for: Impact and cost-effectiveness of different vaccination strategies to reduce the burden of pneumococcal disease among elderly in the Netherlands
Source: PLoS One. 2018 Feb 9;13(2):e0192640. doi: 10.1371/journal.pone.0192640 (PMC5806887; doi:10.1371/journal.pone.0192640)
Supplement: S1 File — (PDF) [file pone.0192640.s003.pdf]

File S1

Main formula in the static model

The post vaccination incidence is calculated by multiplying the pre-vaccination incidence with 1 minus the vaccine-efficacy multiplied by the coverage of vaccination. See formula 1.

$$\text{Projected incidence vac}_{\text{d,t,a,y}} = \text{Projected incidence}_{\text{d,t,a,y}} * \left(1 - \text{Vaccine Efficacy}_{\text{d,t,a}} * \text{coverage}_{\text{t}}\right) \qquad [1]$$

In which d stands for disease type, thus IPD or pneumonia, t for vaccine-type, thus PCV7, PCV10 minus PCV7, PCV13 minus PCV10, PPV23 minus PCV13, non-vaccine types, a for age in years and y for calendar year, thus 2018 until 2027. Future disease incidence was projected as described in the main text.
